# Enriched-GWAS and Transcriptome Analysis to Refine and Characterize a Major QTL for Anaerobic Germination Tolerance in Rice

**DOI:** 10.3390/ijms22094445

**Published:** 2021-04-24

**Authors:** Hedia Tnani, Dmytro Chebotarov, Ranjita Thapa, John Carlos I. Ignacio, Walter K. Israel, Fergie A. Quilloy, Shalabh Dixit, Endang M. Septiningsih, Tobias Kretzschmar

**Affiliations:** 1 International Rice Research Institute, DAPO Box 7777, Metro Manila 1301, Philippines; hediatnani0@gmail.com (H.T.); d.chebotarov@irri.org (D.C.); ignacio.8@osu.edu (J.C.I.I.); W.Israel@westernsydney.edu.au (W.K.I.); f.quilloy@irri.org (F.A.Q.); s.dixit@irri.org (S.D.); 2 Laboratory of Bioinformatics, Biomathematics and Biostatistics, Institut Pasteur de Tunis, Université Tunis El Manar, Tunis 1002, Tunisia; 3 Department of Soil and Crop Sciences, Texas A&M University, College Station, TX 77843, USA; rt485@cornell.edu; 4 Section of Plant Breeding and Genetics, School of Integrative Plant Sciences, Cornell University, Ithaca, NY 14853, USA; 5 Department of Horticulture and Crop Science, Ohio Agriculture Research and Development Center, The Ohio State University, 1680 Madison Ave, Wooster, OH 44691, USA; 6 Southern Cross Plant Sciences, Southern Cross University, 1 Military Road, Lismore 2480, Australia; tobias.kretzschmar@scu.edu.au

**Keywords:** anaerobic germination, *AG2*, candidate genes, enriched haplotype GWAS, rice (Oryza sativa), transcriptomics

## Abstract

Tolerance of anaerobic germination (AG) is a key trait in the development of direct seeded rice. Through rapid and sustained coleoptile elongation, AG tolerance enables robust seedling establishment under flooded conditions. Previous attempts to fine map and characterize *AG2* (*qAG7.1*), a major centromere-spanning AG tolerance QTL, derived from the indica variety Ma-Zhan Red, have failed. Here, a novel approach of “enriched haplotype” genome-wide association study based on the Ma-Zhan Red haplotype in the *AG2* region was successfully used to narrow down *AG2* from more than 7 Mb to less than 0.7 Mb. The *AG2* peak region contained 27 genes, including the *Rc* gene, responsible for red pericarp development in pigmented rice. Through comparative variant and transcriptome analysis between AG tolerant donors and susceptible accessions several candidate genes potentially controlling *AG2* were identified, among them several regulatory genes. Genome-wide comparative transcriptome analysis suggested differential regulation of sugar metabolism, particularly trehalose metabolism, as well as differential regulation of cell wall modification and chloroplast development to be implicated in AG tolerance mechanisms.

## 1. Introduction

Rice is the staple food for more than half of the world’s population; however, the overall rice agri-food sector is rapidly changing, particularly in the developing world [1,2]. On the production side, changing climates exacerbate abiotic and biotic limitations in existing rice-growing areas, while new areas at increased latitudes and altitudes are becoming available for rice cultivation [3,4]. Significant socio-economic changes, such as urbanization and rural industrialization, in conjunction with the water, energy, and food security nexus are major forces driving mechanization and the shift from transplanting to direct seeding (DS) in rice cultivation.

Transplanted rice is one of the most resource-intensive crops in water and labor demand [5]. Yet the supply of water and labor is limiting and becoming increasingly erratic and unreliable, thus challenging traditional cultivation methods. There is substantial opportunity for change, particularly in new areas opening up for cultivation, provided management systems and adapted cultivars are available. Over the last decade, DS rice has become increasingly popular across Asia due to its low-input demand and labor and cost savings [5,6]. One of the world’s leading rice producers, India, now has more than 50% of its rainfed production under DS and Thailand, one of the leading rice exporters, has most of its rainfed rice under DS conditions.

Mechanization of rice cultivation is paramount to fully capitalize on the cost savings potential of DS. While the development of direct seeding technology and agronomy has made substantial progress in recent years [6,7,8], the development of *indica* varieties suitable for DS has lagged behind. Poor germinability and poor crop establishment, with downstream effects on weed competition and ultimately yield, are major challenges that must be addressed for widespread adoption of DS. Direct seeded varieties need to be supplied with a suite of specific traits, most of which relate to uniform and vigorous crop establishment under conditions that drastically differ from nursery establishment and transplanting.

Tolerance of anaerobic germination (AG), the ability of vigorous germination and heterotrophic growth under complete submergence, is among the best-studied traits in regard to DS variety development. Tolerance of AG is a unique and variable trait of rice [9]. Unlike other cereals, rice is adapted to growth in wetland conditions through rapid germination in waterlogged soils and sustenance of heterotrophic coleoptile growth to reach the water surface and transfer oxygen to the seed, thus allowing subsequent growth of the radicle and leaf [10]. However, due to the absence of positive selection under transplanted conditions, AG tolerance has become a rare trait.

DS rice benefits from high AG tolerance irrespective of whether the crop is grown in a rainfed or irrigated environment. Rainfed crop cycles usually coincide with the wet or monsoon season when heavy rains cause temporal flooding. Under irrigated conditions, shallow flooding is preferred to suppress the growth of weeds, which are one of the major constraints in DS conditions.

AG tolerance is a rare trait among tropical *indica* rice, the main rice subspecies grown in South and South-East Asia, and absent from most high-yielding modern *indica* varieties, such as IR64 and IR42 [11]. The screening of over 8000 gene bank rice accessions and breeding lines at the International Rice Research Institute (IRRI) identified nearly two dozen AG tolerant genotypes, including Ma-Zhan Red (MR), Khao Hlan On (KHO), Nanhi, Kharsu 80A [11,12,13,14], and TKM9 [15]. While KHO, MR, and TKM9 are tropical *indica* landraces, Nanhi and Kharsu 80A belong to the *aus* group.

Several major QTLs for AG tolerance were identified among bi-parental populations between donor accessions and highly susceptible popular modern varieties. The cross derived from KHO, a landrace from Myanmar, and the recurrent parent IR64 identified a major QTL on chromosome 9, *qAG-9-2* (referred to as *AG1*). The gene underlying this QTL was cloned as *OsTPP7*, a trehalose-6-phosphate phosphatase gene, which enhances anaerobic germination tolerance [16]. This QTL has been used for crop improvement and has been successfully validated in the field [17,18,19,20,21].

*AG1,* however, remains the only AG QTL that has been fine mapped and characterized at the gene level. Other major AG QTLs have yet to be refined to further our knowledge of the underlying mechanisms of AG and develop predictive marker sets. A priority candidate is *qAG7.1* (*AG2*), a major QTL on chromosome 7, spanning the centromeric region. It was identified in a mapping population derived from IR42 and MR [12], with the favorable allele contributed by the tolerant donor, Ma-Zhan Red. Importantly, this QTL co-localized with QTLs from previous studies, *qAG7.1*, derived from KHO [11], and a QTL derived from TKM9 [15]. Furthermore, it partially overlapped with *qAG7* and *qAG7.1*, derived from Nanhi and Kharsu 80A, respectively [11,13,14]. A genome-wide association study (GWAS) using an *indica* rice panel also detected an AG-associated region in a similar location as *AG2* [22].

Mainly due to poor recombination within the *AG2* region and inefficiency in developing NILs, traditional fine-mapping approaches failed, necessitating the development of an alternative strategy. In this study, we used a diverse panel enriched for potentially AG-tolerant haplotypes at the *AG2* QTL region selected from the 3K Rice Genomes (3K RG) Project [23] to narrow down the QTL through GWAS. In addition, global expression profiling under anaerobic conditions for selected accessions with contrasting phenotypes was performed to identify potential candidate genes and metabolic mechanisms underlying *AG2*.

## 2. Results

### 2.1. AG2-Enriched Panel Developed Based on SNP Variation between Ma-Zhan Red and IR42

Previously, a QTL mapping study from a cross between IR42 and Ma-Zhan Red (MR) identified a major QTL *qAG7.1* (*AG2*) in the region of 6 Mb to 13 Mb on chromosome 7 (LOD = 14.5; R^2^ = 31.7%) [12], which overlapped with several other AG QTLs identified in the region (Figure S1). To narrow down the *AG2* region and identify potential candidate genes, a GWAS approach using an *AG2* haplotype enriched panel was carried out based on data from the 3000 Rice Genomes (3K RG) [24]. However, since known *AG2* parental donors and a susceptible recipient were not included in the 3K RG data set, we re-sequenced them first. Notably, with the exception of TKM9, all *AG2* donors displayed red pericarp, indicative of a functional *Rc* allele. A total of 45,784,570–156,947,986 raw reads were obtained for each MR, KHO, Kharshu 80A, Nanhi, TKM9, and IR42, of which 94.5–97.3% successfully mapped to the Nipponbare reference (MSU7) (Supplementary File S1).

**Figure 1:**
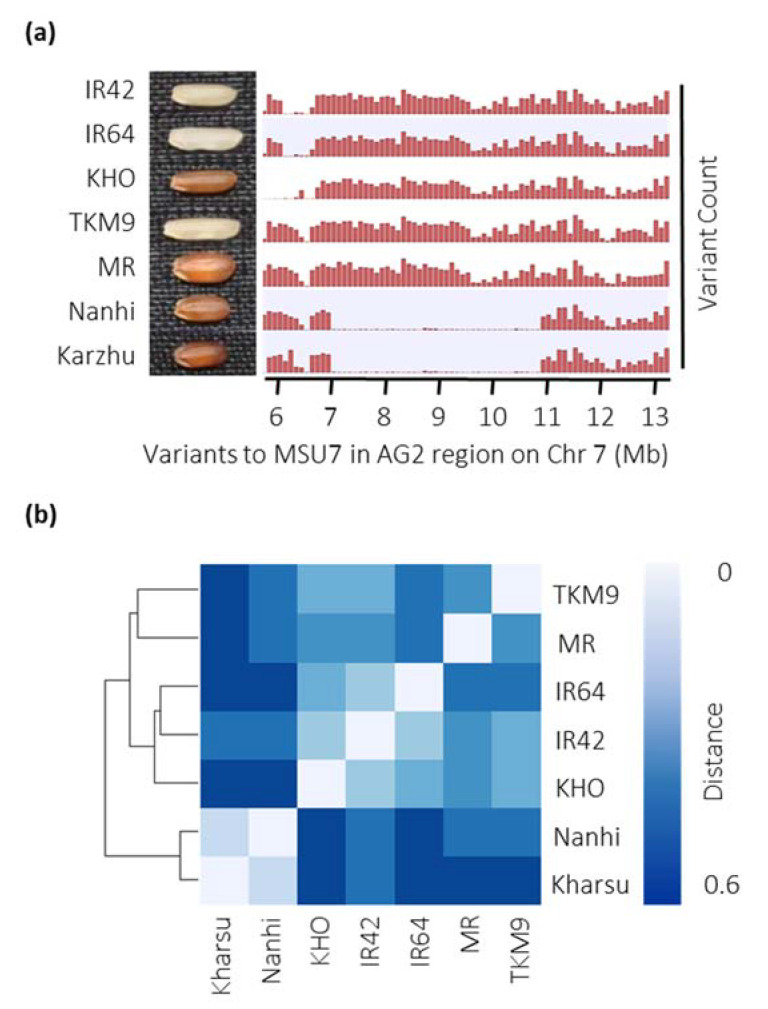
Sequence variation within *AG2* region. (**a**) Sequence variants within the *AG2* region (6 Mb to 13 Mb on chromosome 7) of AG donors (Nanhi Ma-Zhan Red (MR), Khao Hlan On (KHO), Kharshu 80A, and Nanhi) and AG susceptible varieties (IR64 and IR42) relative to Nipponbare. (**b**) Genetic distance (identity-by-state) matrix between AG donors and AG recipients based on variations within the *AG2* region.

Among the donors and recurrent parents, comparative variant analysis across the larger *AG2* sequenced genomic region (6 Mb to 13 Mb) revealed patterns of SNP variation (Figure 1a). The analysis showed that the two *aus* accessions Nanhi and Kharsu 80A shared high similarity with a genetic distance of 0.1 (Figure 1b; Supplementary File S2) and were distant from all other *indica* (average distance of 0.49 for Kharsu 80A and 0.47 for Nanhi). However, in the region between 7 Mb and 11 Mb, they showed minimal SNP variation to the *japonica* Nipponbare reference genome (Figure 1a). Likewise, the two elite AG susceptible *indica* recipients IR42 and IR64 were similar, with a genetic distance of 0.22 (Figure 1b; Supplementary File S2). Interestingly, the *indica* AG donor KHO clustered with IR42 (distance 0.22) and IR64 (distance of 0.26), rather than MR (distance of 0.37) or TKM9 (distance of 0.31). The two AG-tolerant donor *indica* accessions MR and TKM9 were not very similar, with a distance of 0.35. Overall, the region discriminated better between the subspecies *aus* and *indica* than the AG phenotype. Considering that the IR42/MR population had the largest effect *AG2* QTL at the highest physical resolution (Figure S1), we decided to focus on variation between these two genotypes for downstream analysis.

Within the *AG2* region (6 Mb to 13 Mb on chromosome 7), 16,193 SNPs showed polymorphism between MR and IR42 and were used as the enriched panel selection criteria (as described in M&M; Figure S2a). A subset of 351 accessions from the 3K RG was selected as an enriched *AG2* panel. Notably, the selected accessions were rather diverse as to being MR-positive (being among the 100 closest matchings to the MR haplotype in the respective bin) or MR-negative (not among the top 100 matching with MR bin haplotype) between bins for the first half of the region (9 bins). In contrast, the second half of the region was relatively conserved, either being MR-positive or MR-negative across the region (8 bins) (Figure S2b). This seems to indicate a lack of ancestral recombination in the downstream half, which is in line with the centromeric region of chromosome 7 predicted to be located at around 12.1 Mb (http://rice.plantbiology.msu.edu/annotation_pseudo_centromeres.shtml; accessed on 9 September 2019). The panel consisted of 62.4% (219) accessions with white pericarp, 30.4% (107) red colored pericarp, and 7.1% (25) no observation recorded for pericarp color (Figure S3). Most accessions were from East Asia (126), South Asia (108), and South East Asia (87); while the rest were from Africa (13), America (10), Middle East (1), and unknown (6). They were mostly *indica* (93.7%) and some *aus* (5.4%) (Supplementary File S3).

### 2.2. Significant Phenotypic Variation Displayed within the Enriched-AG2 Rice Panel

The selected enriched panel of 351 accessions showed significant variability of survival rate. The phenotype distribution was skewed to the left, toward AG susceptibility (Figure S4a, left panel). The survival rates of IR42 and MR were 0.8% and 34%, respectively. It was noted that even the tolerant donor survival rates were also not as high as previously reported (IR42: 3.3%, MR: 63%) [12], which was likely due to high temperature (max of 39 °C) during the AG screening. Due to the data skewness, the phenotype data were transformed using the logit transformation prior to GWAS [25]. The phenotypic distribution of the transformed data was improved (Figure S4a, right panel). The Q-Q plot of the logit-transformed data was much closer to normal (Figure S4b). Regressing the transformed phenotype on the first 5 principal components yielded normally distributed residuals with Shapiro–Wilk test accepting normality (*p* = 0.8) (Figure S4c, right panel).

Most of the accessions with colored pericarp exhibited the functional allele for *Rc*. In contrast, most of the accessions with white pericarp contained the 14bp deletion in the coding region of *Rc*, indicative of a non-functional allele (Supplementary File S4). Interestingly, several accessions having a colored pericarp did not share the functional allele. Likewise, several of the accessions having white pericarp possessed the functional allele, including the AG donor TKM9 (Supplementary File S5).

### 2.3. GWAS of the Selected Enriched Panel Significantly Narrowed down the AG2 Region

**Figure 2:**
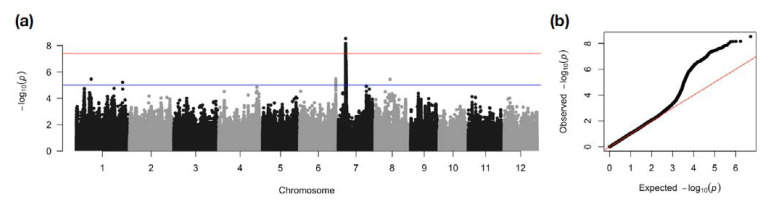
Detection of *AG2* peak on chromosome 7 in the enriched GWAS panel. (**a**) The Manhattan plot shows the level of significance for SNPs correlated with AG tolerance from the GWAS analysis (numbers across the x-axis indicate the chromosome). (**b**) The QQ plot is shown at the right. The blue line indicates the *p* < 0.00001 threshold and the red line indicates the Bonferroni correction.

To assess the *AG2* enriched panel for the presence of AG QTLs and to narrow down the region of *AG2*, a GWAS was performed using both filtered and linkage disequilibrium (LD) pruned SNP-genotype dataset with the transformed data of the survival rate. It revealed a single peak with negative log *p*-values of up to 8.531 at around 6 Mb to 6.7 Mb on chromosome 7, which passed the Bonferroni-corrected significance threshold (Figure 2). GWAS was also conducted for pericarp color using the enriched panel, and a single dominant peak on chromosome 7 confirmed the previous report of the *Rc* gene as a major contributor to the red pericarp trait (Figure S5). The red pericarp peak overlapped with the AG peak of the panel, covering a region between 5.6 Mb and 6.8 Mb (Figure S6).

### 2.4. Five Major Haplotype Groups Identified within the Peak Region of AG2

**Figure 3:**
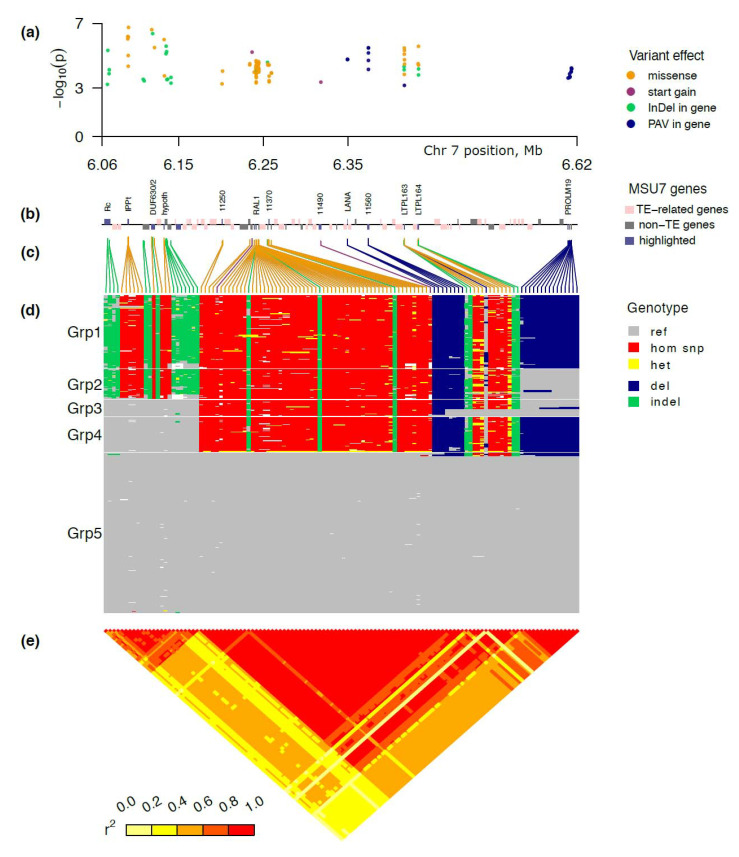
A close-up view of *AG2* peak region on chromosome 7. (**a**) The Manhattan plot shows the level of significance for SNPs correlated with AG tolerance from the GWAS analysis (numbers across the x-axis indicate the chromosome). (**b**) The gene track identified within the region (above line—positive strand, below line—negative strand, light red—transposable elements, genes marked blue are some of the selected candidate genes). The annotation is taken from MSU7, numbers like 11560 mean LOC_Os07g11560. (**c**) Lines connecting physical position plots (**a**,**b**) with consecutive variant plots (**d**,**e**). The color of lines shows the effect (orange—nonsynonymous SNP, green—small indel, blue—large deletion or its part). (**d**) Graphical genotype plot (columns are variants, rows are samples, genotypes are color-coded according to legend in the bottom; the samples are ordered and grouped by hierarchical clustering). (**e**) LD heatmap of the region using r^2^.

The peak region contained 4016 SNPs and 1029 INDELs. Among the SNPs, 392 were missense, 239 synonymous, 19 stop gain, 5 start gain, 1 start lost, 2 splice donor, 2 splice acceptor, 523 intron variants, and 20 splice region variants. Together with 277 deletion clusters from Fuentes et al. dataset [26] and 4469 PAV (presence-absence variations, see methods), the total region variant dataset comprised 9791 variants and a mixed-model GWAS with the same settings as before was run on this dataset (with the same kinship matrix and covariates). The most significant SNP was positioned at 6,086,998 bp, having a *p*-value of 8.531 in the intergenic region upstream of LOC_0s07g11050 (IPP transferase, putative expressed) and around 20 kb downstream of LOC_Os07g11020 (Rc, the bHLH transcription factor regulating proanthocyanidin production in seeds) (Figure 3a,b; Supplementary File S6).

To simplify visualization and downstream analysis, we focused only on variants that: (1) are located in gene regions (or overlap gene regions, for INDELs and PAV); (2) have minimal significance level –log10(*p*) > 3; and (3) leave only SNPs that are predicted to have an effect on protein sequence or splicing (missense, start/stop gain or loss, and splice donor or acceptor variants), as well as gene region INDEL and PAV (Figure 3c). After this filtering, we were left with 120 variants in the region 6.065 Mb to6.625 Mb (Supplementary File S7): 73 missense SNPs, 2 start gain SNPs, 21 INDELs, and 24 presence-absence variations (Figure 3). The reduced Manhattan plot showed that signals of highly significant genic SNPs INDELs and PAV were present throughout the 6.0 Mb to 6.7 Mb region, with variants of highest significance found in the proximal part of the *AG2* region (Figure 3a).

Within the MSU7 reference genome, the narrow *AG2* region of 6.0 Mb to 6.7 Mb contained 27 annotated genes (including expressed and hypothetical proteins) and 52 transposable elements (TE) (including retrotransposons) (Figure 3b; Supplementary File S6). Among the annotated genes were several seed storage proteins, including six seed allergenic proteins (RAL1 to RAL6), three prolamins (PROLM 19, 20, and 22), and three protease inhibitor/LTP family proteins. In total, three loci were annotated as regulatory proteins, a leucine zipper protein (LOC_Os07g10970), Rc (LOC_Os07g11020) [27], and a DUF630/DUF632 domains containing protein (LOC_Os07g11070) annotated as a putative basic leucine zipper (bZIP) protein.

Nearly all of the annotated genes contained protein-altering variants potentially associated with the phenotype (*p* < 0.0005, Supplementary File S6), with the most significant among those, a missense SNP, found in an IPP transferase (LOC_Os11050). Notably, the DUF630/DUF632 domains containing protein (LOC_Os07g11070) had two missense variations and one INDEL, which were among the highest significant variations in the *AG2* peak region (Figure 3). The leucine zipper protein (LOC_Os07g10970) and a kinase-associated phosphatase (LOC_Os07g11010) upstream of Rc did not contain any significant protein-altering variants.

Simplified haplotype structure revealed 5–6 major blocks with near-perfect LD, each having essentially two distinct alleles (Figure 3d,e). The blocks were tightly linked to each other, such that combinations of these block alleles defined only five major haplotype groups (Grp 1–5) across the whole peak region (Figure 3d and Figure S7a). Further, the phenotypic distribution of the transformed haplotype groups was improved (Figure S7b).

Modeling phenotype by haplotype groups using binomial GLM showed that in each of the haploblocks the effect was significant (−log10(*p*) > 10; Supplementary File S8). Arranging phenotypes by haplotype groups (Figure S7) revealed that Grp 1, with mostly alternative alleles to the Nipponbare reference including wild-type functional Rc and MR-type DUF630/DUF632 domains containing protein (LOC_Os07g11070), had the highest average rates of survival, followed by Grp2, with mostly alternative alleles except for the prolamin block towards the end of the region. Interesting to note was that the recombinant haplogroups (Grp3 and Grp4) had a lower average phenotype that the reference-allele Grp5. While MR and Nanhi belonged to Grp1, Kharsu 80 and TKM9 belonged to Grp2, consistent with above-average survival rates. Interestingly, KHO, like IR42 and IR64, belonged to the reference Grp5 with below-average survival rates under AG. While this did not fit with KHO being an *AG2* donor, it was consistent with genetic distance analysis across the *AG2* region (Figure 1b).

### 2.5. Significant Expression Patern Differences between Tolerant Donors and Susceptible Parental Genotypes Revealed by RNA-Seq Analysis

To further characterize the candidate genes in the refined *AG2* region and provide insight into global expression profiles of accessions with contrasting phenotypes under AG, RNA-Seq analysis was performed on four tolerant genetic donors and two susceptible accessions. An average of 18.78 ± 1.38 million clean reads were generated per sample, 89.2% to 95.3% of which were successfully mapped to the MSU7 reference genome. The detailed summary for these cDNA libraries is presented in Supplementary File S9.

Based on an FDR corrected *p*-value < 0.05, pairwise comparisons between differentially expressed genes (DEGs) of the four tolerant lines, MR, KHO, Kharsu, and Nanhi, relative to IR42 and IR64 were made (Supplementary File S10). The union between all DEGs within the 8 pairwise comparisons and filtering of significant DEGs for presence in at least 2 pairwise comparisons resulted in a list of 20,212 loci (Supplementary File S11). Of these 17,912 (89%) successfully mapped to 1114 bins in MapMan, of which 17 were ranked as significant (Supplementary File S12).

**Figure 4:**
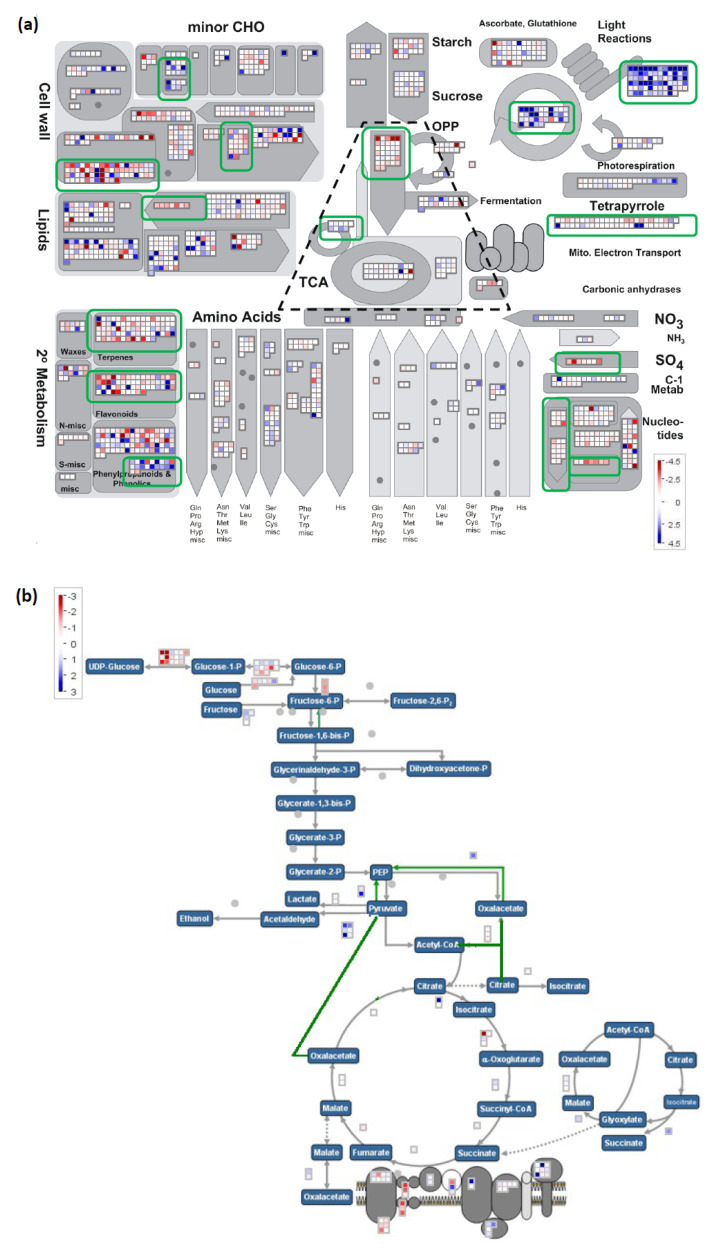
Mapman visualizations of the union of 20,212 (17,938 successfully mapped) differentially expressed genes (DEG) (FDR-corrected *p* < 0.05) of 8 pairwise comparisons between four AG tolerant lines (Nanhi Ma-Zhan Red, Khao Hlan On, Kharshu 80A, and Nanhi) and two AG susceptible varieties (IR64 and IR42). (**a**) Metabolism overview map representing 1724 DEG. DEG upregulated in AG tolerant lines are represented in blue boxes and downregulated DEG in red boxes. Grey circles indicate the absence of DEG in these bins. Black dotted trapezoid indicates pathways highlighted in (**b**). Green rounded rectangles indicate significant bins. (**b**) Glycolysis and TCA cycle map representing 124 DEG. DEG upregulated in AG tolerant lines are represented in blue boxes and downregulated DEG in red boxes. Grey circles indicate absence of DEG in these bins.

Significant bins included photosynthesis, cell wall modifications, carbohydrate metabolism, secondary metabolism, nucleotide metabolism, and fatty acid desaturation. All are represented in the metabolism overview (Figure 4a). Together significant bins comprised 447 unique loci out of the 1724 loci represented in the metabolism overview. The most significant bins belonged to light reaction and Calvin Cycle, with most DEG in these bins found upregulated in AG tolerant lines. This seemed surprising given that the tissues consisted of white coleoptile and remnant embryo tissue, devoid of any signs of chlorophyll, harvested 4 days after imbibition in the dark under complete submergence, conditions prohibitive of photosynthesis.

There were two significant bins with DEGs exclusively upregulated in AG tolerant lines associated with trehalose metabolism and contained *OsTPP7*, the causal gene of the *AG1* QTL, which was consistently among the most upregulated DEG across all four AG tolerant lines (Supplementary File S11). The two large significant bins, corresponding to cell wall modification and cell wall degradation, showed mixed differential expression with a near equal amount of down and upregulated entries in AG tolerant lines, suggesting differences in cell wall dynamics between AG tolerant and susceptible lines. Then, three bins associated with secondary metabolism, isoprenoids and terpenes, flavonoids, and simple phenols, showed mixed differential expression. While the former two contained largely downregulated DEGs in AG tolerant lines, DEGs associated with simple phenols were mostly upregulated in AG tolerant lines. Significant bins mostly had DEGs downregulated in AG tolerant lines related to sulfur metabolism and nucleotide metabolism.

A closer look at glycolysis, TCA, and energy metabolism (Figure 4b) revealed differences apart from the significant glycolysis and glyoxylate cycle bins (Figure 4a). DEGs in glycolysis were mostly related to glucose-1-P conversion to either UDP-glucose or glucose-6-P. No DEGs were found in the pathway leading from fructose-6-P to PEP. TCA-related and glyoxylate cycle DEGs were moderately upregulated in AG tolerant lines. Upregulated DEGs in fermentation were largely associated with the conversion of pyruvate to acetaldehyde, suggesting enhanced alcoholic fermentation in AG tolerant lines.

### 2.6. Four Distinct Clusters Identified by Regional RNAseq Analysis of the Peak Region of AG2

**Figure 5:**
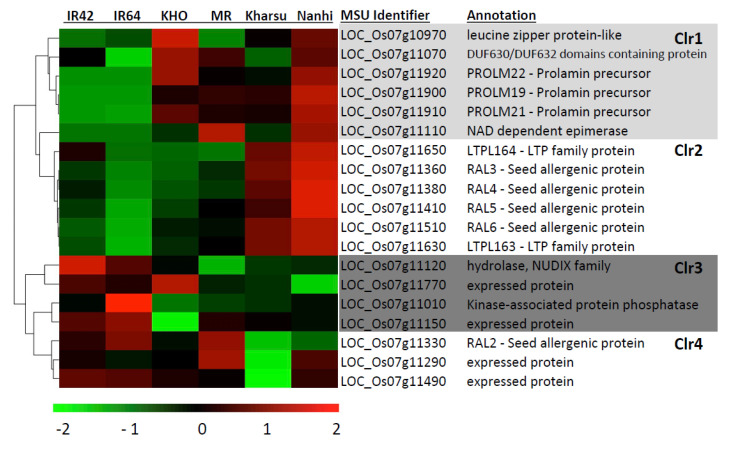
Heat map and the hierarchical clustering of differentially expressed genes (DEG) within the *AG2* peak region. The heat map shows the log2FC of relative gene expression within the region across all six accessions. The default transformation method, rlog of deseq2 package [28] was used to transform the gene count output from StringTie (v.2.1.4). The rlog transformed value of gene counts scaled by library size was used to plot the heatmap. Red indicates up-regulation, green shows down-regulation, and black is neither up- or down-regulated. Clr = cluster.

Of the 27 genes in the *AG2* region, 19 were expressed at detectable levels (Figure 5). Interestingly, *Rc* and the *IPP transferase* in the proximal region that showed the highest significant variants (Figure 3) were not expressed. Heatmap visualization of normalized gene counts for all DEGs across two AG susceptible and four AG tolerant lines revealed four distinct clusters (Figure 5). Cluster1 (Clr1) contained genes relatively highly expressed across the AG tolerant lines. Clr1 included the leucine zipper regulatory protein, three prolamin genes (PROLM 19, 21, and 22), an NAD-dependent epimerase, and the DUF630/DUF632 domains containing protein (LOC_Os07g11070). Cluster 2 (Clr2) genes were more highly expressed only in the *aus* donor lines (Kharsu and Nanhi) and included seed allergenic proteins and LTP protein. Cluster 3 (Clr3) genes were consistently more highly expressed in susceptible lines relative to the donor lines. They included a kinase-associated protein phosphatase, a hydrolase and two expressed proteins. Cluster 4 (Clr4) genes were less expressed in the 2 *aus* lines and included a seed allergenic protein and two expressed proteins.

## 3. Discussion

Bi-parental QTL mapping approaches using various AG trait donors have consistently identified *AG2* (Figure 1) [11,12,13,14,29]. LOD scores for *AG2* QTLs from the bi-parental mapping approaches were high (Figure S1), and the variance explained ranged from 9.9% to 39.7%. *AG2.* However, the physical size of the *AG2* QTLs was quite large, spanning a region from 5 Mb to 18 Mb (Figure S1). Collectively these results supported the presence of *AG2* in a variety of genetic backgrounds but did not enable precise localization of the *AG2* causal mutation for downstream trait marker development or candidate gene approaches.

In most cases, the *AG2* QTL region spanned the predicted centromeric region, where recombination events are reportedly lower [30], likely contributing to the poor resolution of *AG2* in QTL mapping. One GWAS result identified a QTL for AG on chromosome 7 that was proposed to be overlapping with *AG2*; however, the *p*-values were relatively low [22]. This might partly be due to poor representation of *AG2* in their selected panel, poor ancestral recombination in the region, or a combination of both factors. Moreover, their chromosome region spanning from 17.9 Mb to 18.3 Mb does not match the overlapping regions of the previous biparental QTLs, that largely ranged from 6 Mb to 13 Mb (Figure S1), suggesting the possibility of more than one QTL on chromosome 7.

Enrichment of GWAS panels can be achieved based on phenotype or genotype information. Since we specifically targeted the *AG2* region, we decided on a latter approach. To our knowledge, enrichment of GWAS panels based on genotype data has not been previously described in crops. We used SNP information within the MR *AG2* region to screen the 3K RG panel for related germplasm. Based on a new comparison of the overlapping regions of the *AG2* QTLs (Figure S1), we focused on a region spanning 6 Mb to 13 Mb. Rather than using the whole *AG2* region, we arbitrarily divided the region into 17 bins of ~1000 SNPs and used the respective MR-SNP haplotype of each region to query the 3K RG. This strategy had several advantages: (1) higher resolution power since all the SNPs were accounted for regardless of the physical size of each bin; (2) removal of bias against potentially highly conserved regions that might mask less conserved regions; (3) anticipating that there might be two or more sub-regions within *AG2* that could contribute to the observed phenotype in MR, but that might not be co-segregating in other genetic backgrounds.

The final GWAS panel contained mainly *indica* accessions (329), a smaller number of *aus* accessions (19), and several admixed accessions (3) (Figure S3; Supplementary File S3), confirming that *AG2*-related genotypes exist in both sub-groups (MR and KHO are *indica*, while Nanhi and Kharsu are *aus*). Interestingly, even though the panel was highly enriched for potential MR-derived *AG2* haplotypes, phenotyping revealed that most accessions were not AG tolerant. Phenotype distribution was heavily skewed toward low AG survival rates. MR was still among the most tolerant accessions, indicating that the causal *AG2* variation is either rare (Figure S4) or masked in specific genetic backgrounds. Nevertheless, a single significant QTL peak for AG encompassing the Nipponbare reference region of 6 Mb to 6.7 Mb on chromosome 7 was detected using the enriched GWAS analysis within the *AG2* region, effectively refining the *AG2* QTL by approximately 10-fold.

Interestingly, when the same panel was used to detect associations with red pericarp, a single highly significant peak at 5.6 Mb–6.8 Mb on chromosome 7 was detected (Figure S5; Figure S6); largely congruent with the AG peak. The proximity of the AG peak marker to *Rc* and the tight overlap of AG and red pericarp GWAS peaks within the enriched panel suggested a tight linkage of both traits or even possible pleiotropy of Rc. This was supported by (1) a significant correlation of red pericarp and AG tolerance, and (2) the finding that all known *AG2* trait donors feature a red pericarp phenotype, with the exception of TKM9 (Figure 1a).

Genome-wide comparative expression analysis of four AG tolerant lines vs. two AG susceptible lines supported previous findings that AG tolerant accessions are able to deal with AG-conditions through maintenance of active metabolism, driving growth through cell expansion by efficient breakdown of storage carbohydrates, while mitigating the deleterious effects of anaerobic energy metabolism (alcohol fermentation) through alcohol catabolism and antioxidant activity (Figure 4, Supplementary File S12). Notably, upregulation of trehalose metabolism, including upregulation of the *AG1* gene *OsTPP7,* was among the significant pathways distinguishing AG tolerant lines from susceptible ones, underlining the importance of trehalose-6-phosphate mediated source sink regulation during AG [16]. Since both *AG1* and *AG2* are present in all four AG tolerant lines, it was not possible to distinguish between AG- and *AG2*-specific effects in our set and it is quite possible that some of the observed differential expression between tolerant and susceptible lines is due to the presence-absence of *OsTPP7*.

Surprisingly, light reactions, Calvin cycle and tetrapyrrole biosynthesis were among the significantly upregulated pathways in AG tolerant lines, even though anaerobic germination was performed in complete darkness for 4 days. Thus, active photosynthesis was not possible, even in the tolerant accessions and the observed transcript signature indicative of photosynthesis, thus reflecting an overall preparation of the tolerant genotypes to switch from heterotrophism to autotrophism once light and air are available. We speculate that the surplus energy and carbon available to AG tolerant lines under AG is thus not only invested in cell expansion to expand the submerged coleoptile towards light and air, but also to drive etioplastic differentiation even though tissues were fully white at times of harvest and did not show any signs of chlorophyll.

Among the 27 annotated genes within the *AG2* peak region, 19 were differentially expressed between AG-tolerant and AG-susceptible genotypes under AG conditions (Figure 5). *Rc*, however, was neither expressed in AG-tolerant nor in AG-susceptible genotypes at detectable levels. This speaks against pleiotropic effects of *Rc* being responsible for the observed AG phenotypes unless *Rc* is transcribed earlier on during germination. Published AG transcriptomic studies do not support *Rc* expression, even at earlier time points [31,32,33,34]. Consequently, there is no evidence supporting the transcription of Rc during germination or vegetative growth. *Rc* is mainly expressed in panicles during grain filling stages [27], where it orchestrates the accumulation of proanthocyanidins (PA) in the developing pericarp that gives red rice its distinct coloration. While flavonoid biosynthesis was among the significant bins in MapMan analysis, DEGs within the bin were mainly downregulated in AG tolerant backgrounds, which further argues against Rc activity during germination.

It could simply be that *Rc*-mediated differences in PA concentrations in the pericarp contribute to the observed differences in AG tolerance. PAs have been demonstrated to exhibit strong antioxidant properties [35,36,37] and could influence hypoxia-related stress signaling or mitigate the hypoxia-related accumulation of ROS during germination. It was reported that PAs regulated some key enzymes that control the levels of ROS and the antioxidant capacity in the germinating seeds of *Arabidopsis* [38] and antioxidant activity and response to oxidative stress are among the enriched GO’s of AG donors (Supplementary File S12). However, the finding that not all red accessions of the panel were tolerant and that the highly AG tolerant TKM9 did not exhibit PA accumulation in the pericarp seems to argue against this. Consequently, while *Rc* cannot be entirely ruled out as a candidate gene, it is unlikely, and further studies in this respect are warranted.

In total, three prolamins, PROLM19, PROLM 20, and PROLM22, were highly upregulated in all four AG tolerant genotypes relative to the two AG susceptible ones (Figure 5). Prolamins are seed storage proteins of cereals synthesized during grain filling [39]. After glutelins, they are the most abundant storage proteins in rice [39], and Nipponbare (MSU) contains 28 putative prolamins. Expression of prolamins during late germination has not been specifically reported, but all three genes were also upregulated under AG in an earlier transcriptomic study by Narsai et al. [33]. Interestingly, PROLM 24 (LOC_Os06g31070) was previously suggested as a biomarker for successful seed priming in rice, supporting a yet unidentified role for prolamin synthesis during germination [40]. However, the finding that Grp1, the haplogroup showing the highest average survival rates, largely showed the absence of these three prolamins argues against these genes being causal for the *AG2* phenotype.

A leucine zipper protein (LOC_Os07g10970), in close proximity to *Rc* and the most significant SNP, was also relatively more highly expressed in all AG tolerant varieties, except MR (Figure 5). However, it was highly conserved, and no protein-altering variants were found within the panel. The DUF630/DUF632 domains containing and putative bZIP protein (LOC_Os07g11070) were more highly expressed in all AG tolerant lines except Kharsu (Figure 5). In Grp1 and Grp2, which were the two haplotypes associated with higher survival under AG, LOC_Os07g11070 did show several protein-altering variants, with the potential to affect function. As a putative regulatory protein, differentially responsive to AG in AG tolerant lines and with protein-altering variants associated with high AG survival, LOC_Os07g11070 seemed a likely candidate to explain the global downstream effects of the *qAG2* region on gene expression and phenotype. A dysfunctional DUF630/DUF632 domains containing protein on chromosome 10 in rice, REL2, was implicated with altered cell development and morphology in leaves [41]. The *rel2* mutation was further associated with transcriptional changes of many structural and regulatory genes involved in leaf cell morphogenesis, suggesting a potential regulatory role of DUF630/DUF632 proteins in cell development. Similarly, LOC_Os07g11070 could affect coleoptile cell shape and promote elongation during AG.

Compared to the results of Hsu and Tung [22], this study demonstrates the advantages of enriching a GWAS panel based on genotypic similarity in a target QTL region. It is likely that the “*AG2*” peak at 18 Mb identified by Hsu and Tsung [22] is distinct from the *AG2* QTL at ~6 MB, and it did not appear in our panel since we did not enrich for it. Similarly, neither *AG1* nor any other previously identified AG QTLs was detectable in the enriched panel. Collectively this demonstrates the advantages and disadvantages of using a “haplotype-enriched panel” to delimit the region of a QTL. This is similar to a fine-mapping approach, where a specific focus is given to novel recombination in a selected QTL region, resulting in higher resolution and sensitivity in the target peak region at the expense of other QTLs that might have been detected in the original QTL mapping population. The haplotype-enriched panel offers some advantages over traditional fine-mapping, which include: (i) higher resolution is achieved even in poor recombination regions such as *AG2*; (ii) no time-consuming backcrossing and progeny-testing; and (iii) the original QTL is validated in other genetic backgrounds in the enriched-GWAS panel. A distinct disadvantage, however, is that GWAS activities do not yield in near-isogenic lines (NIL) containing the target QTL to be used for varietal improvement.

## 4. Materials and Methods

### 4.1. Resequencing of Potential Donors and Susceptible Recipients

Resequencing was performed for IR42, IR64, MR, KHO, Kharsu 80A, Nanhi, and TKM9 since they were known *AG2* donors but not part of the 3K RG data set. DNA was extracted using Qiagen DNAeasy Plant Maxi kit (QIAGEN, Germantown, MD, USA), according to the manufacturer’s instructions. Sequencing was performed by Macrogen (Seoul 08511, Korea), generating 100 bp paired-end reads on a HiSeq1000 (San Diego, CA, USA), using TrueSeq technology and lane partitioning via barcoding. Quality control, read mapping and variant calling against the Nipponbare reference genome was performed within the CLC Genomics Workbench 7.0.4 (QIAGEN, Germantown, MD, USA) using the following parameters: Mapping mode: map reads to contigs; Mismatch cost = 2; Insertion cost = 3; Deletion cost = 3; Length fraction = 0.5; Similarity fraction = 0.8. Identity by state (IBS) genetic distance matrix at chromosome 7 from 6 to 13 Mb was calculated based on variants (SNPs and INDELs) from the resequencing data of known AG donors and recipients, replacing missing calls with the reference genotype.

### 4.2. Development of an Enriched-GWAS Panel

An enriched panel consisting of 351 rice accessions was selected from the 3K RG dataset [23] using the following strategy: variants of IR42 and Ma-Zhan Red (MR) relative to the Nipponbare reference within the QTL region (chromosome 7, 6 Mb to 13 Mb) were considered for the selection. A total of 17,611 variants were identified that either show polymorphism between MR and IR42 or, in case the genotype of IR42 was not called, between MR and the Nipponbare reference. Of these, 16,193 variant positions were found in the 3K RG dataset. Based on this list of variant positions, the region was split into 17 blocks of 1000 variants each, except the last one, which contained 193 variants. For each block, we sorted the 3000 varieties from the 3K RG according to the number of positions within the block where the genotype matches that of MR, while the missing calls were counted as a half-match. The top 100 varieties with the highest match count were then selected in each block and assigned as MR-positive. After merging the selections from each block and removing duplicates, we obtained 490 accessions. Of these, 351 accessions from the IRRI gene bank that had available seeds and possessed germination rates higher than 80% under control conditions were used for the GWAS panel.

### 4.3. Phenotyping

Seed germination tests in Petri dishes lined with moist filter paper and incubated in a germination chamber at 30 °C were conducted for quality assessment of the seeds. Germination rate was scored after 4 days after imbibition (dai) and 7 dai. Accessions with rates below 80% were excluded from the panel. Phenotyping was conducted in a screen house at IRRI, following the protocol of Septiningsih et al. [12] in December 2015, February 2016, and March 2016. Dry seeds were sown on trays filled with 1.5 cm of fine soil and covered by another layer of 1 cm. A total of 20 seeds per entry with three replicates per entry in each experiment were used. An alpha plus design was used for randomization, including the two controls (IR42 and Ma-Zhan Red) used in each tray. The trays were then submerged in 8 cm of tap water, and the survival rate was counted on 14 and 21 days after sowing (das), with the latter data used in GWAS.

Since the panel was selected based on haplotypes in a region that covers the domestication gene *Rc* (a gene that is responsible for red pericarp color), pericarp color was also recorded and was used in separate GWAS analysis.

The function glm() from R stats package was used to model survival rate (using binomial family GLM with logit link), and the zero-inflated extension of this model using glmmTMB package was selected [42].

### 4.4. Association Mapping of an Enriched Panel Selection

Genotype data for panel accessions was prepared as follows. The 3K RG Filtered SNP set (4.8M SNPs) was downloaded from the SNP-Seek website (http://snp-seek.irri.org; accesed on 17 August 2018) [43], data for the selected panel were extracted, applying filtering criteria (minor allele frequency > 3%, missing rate < 16%), resulting in a dataset of 2,891,859 SNPs (filtered set). This set was further LD pruned using PLINK v1.90 indep-pairwise command with parameters r^2^ = 0.8, window 20 kb, window step 1, arriving at a set of 231,112 SNPs. The kinship matrix on the LD pruned was set using GEMMA software (command gemma –gk 1). The top five principal components of the linkage disequilibrium (LD) pruned set to be used as covariates were computed using PLINK --pca command [44,45]. The mixed linear model approach was preferred because it takes the relationships among individuals through the kinship (K) matrix and the population structure into account [46,47,48]. Mixed linear model was run using Genome-wide Efficient Mixed Model Analysis software (Genome-wide Efficient Mixed Model Analysis; GEMMA v.0.95a, PLINK v1.90b3.46; http://www.xzlab.org/software.html; accessed on 5 May 2017) [49] on both filtered and LD pruned set, using kinship matrix and top five principal components. The survival rate was used as a primary phenotype. For the subset of samples with a non-zero survival rate (*n* = 295), the same GWAS procedure was run on the logit-transformed phenotype (computed as Y_logit = log(Y/(1 − Y)) where Y is the proportion of survival rate). For the filtered set, the total number of SNPs analyzed by GEMMA was 2,525,802 for the original phenotype and 2,512,003 for the transformed phenotype due to additional filtering within the software defined by default parameters (namely, missing rate <5%). For the additional GWAS for pericarp color, encoded as a binary phenotype (white vs. colored), we used a logistic regression model implemented in PLINK. For peak region analysis, the GWAS was re-run with the extended dataset comprising SNPs, short INDELs, and larger deletions [26].

### 4.5. Peak Region Analysis Using INDELs and Presence-Absence Variations (PAV)

For peak region analysis (chr7 6.0–6.7 Mb), we prepared an extended dataset comprising both SNPs, short INDELs, and larger (>50 bp) deletions [26], and presence-absence variations (PAV) organized as explained below. The INDEL dataset was taken from 3K [24]. For PAV data preparation, each large deletion cluster in the Fuentes et al. dataset consists of deletion calls in different samples that have substantial overlap but may have different breakpoints. While this representation is useful to focus on a unique evolutionary event, it is useful to have a presence-absence variable for every base in the reference for the purpose of association analysis. Thus, we also post-processed the per-sample deletion data to get a presence-absence signal with single-base resolution. The boundaries of these PAVs were computed using the “disjoin“ function in the GenomicRanges package [50] from Bioconductor. For haplotype analysis, we computed a distance matrix based on the filtered variants and clustered the samples into 5 haplotype groups using hierarchical clustering. Linkage disequilibrium (LD) between SNPs was measured as squared correlation (r^2^) using R. To evaluate phenotype differences between groups, we used the ‘compare_means’ function from the R package ‘ggpubr’ [51]. The LD heatmap, genotype heatmap, and other elements of Figure 3 were created using the R package ‘grid’.

### 4.6. RNAseq of AG Donors and Susceptible Parents

Seeds from four AG donors (MR, Khao Hlan On (KHO), Kharsu 80A, and Nanhi) and two AG susceptible parents (IR42 and IR64) were obtained from the IRRI gene bank and stored at 4 °C after post-harvest processing. Seed dormancy was broken by incubation at 50 °C for 5 days. Seeds were de-hulled, sterilized in 70% ethanol for 2 min, washed three times with sterile water, and submerged in beakers with 25 seeds each and 8 cm of autoclaved distilled water for 4 d at 30 °C in a germination chamber in the dark. The coleoptile and remaining embryo tissue were harvested after 4 d and snapped frozen in liquid nitrogen and stored at −80 °C. Each experiment was performed individually and repeated independently four times.

Total RNA was extracted on dissected coleoptiles from the seeds and ground in liquid nitrogen following the method by Li and Trick [52] with some modifications. The clean-up was performed using the RNeasy kit (Qiagen) and then quantified using Nanodrop (ND-1000; Labtech, Paris, France). RNA quality was examined using a 2100 Bioanalyzer (Agilent Technologies, Santa Clara, CA, USA). High-quality RNA samples for library construction were selected based on 260 nm:280 nm ratio and RNA integrity number (RIN) above 8.0. A total of 24 samples were sent to Macrogen, Korea, for sequencing on an Illumina HiSeq2500 platform generating 100 bp paired-end reads.

FastQC [53] was used for the analysis of read quality and its visualization. Low-quality bases and library adapters were removed using Trimmomatic ver. 0.36 [54]. Reads were mapped against Nipponbare (IRGSP build 1.0 RAP-DB) while the mapping and alignment of reads were analyzed using HISAT2 (hierarchical indexing for spliced alignment of transcripts 2) [55]. The DESEQ2 package of Bioconductor was used to test for pairwise differential expression analysis and gene count analysis [28]. To compare gene expression within the *AG2* region, expression levels were quantified by normalizing total gene counts with the effective library size.

For genome-wide expression analysis, pairwise log fold changes of all 4 AG tolerant lines vs. all 2 AG susceptible lines (8 comparisons in total) were filtered for FDR corrected *p*-values below 0.05 and only DEG present in at least 2 out of the 8 comparisons were considered for further analysis. MapMan [56] visualizations against the RAP-DB map were used to visualize the gene expression data.

## 5. Conclusions

An “enriched-haplotype” GWAS approach for *AG2* enabled the narrowing down of the *AG2* region to ~700 kb, where other methods have failed. The *AG2* peak overlapped with a peak for red pericarp coloration and contains *Rc*, a known major modulator for proanthocyanidin accumulation in rice pericarp. While the contribution of *Rc* to the observed AG tolerance phenotype in the panel cannot be ruled out, a strong linkage between non-functional *Rc* alleles and the variation responsible for AG-susceptibility are more likely to be responsible for the observed correlation between red pericarp and functional *Rc* allele and AG tolerance. A strongly Rc-linked DUF630/DUF632 protein with potential regulatory function emerged as a priority candidate gene within the *AG2* region as it showed consistent expression differences and allelic variation between AG tolerant and AG susceptible lines. Strong selection for white pericarp and consequently non-functional *Rc* alleles in rice breeding may have thus inadvertently caused the purging of *AG2* donor alleles from the majority of the available breeding pool.

## Data Availability

All sequencing data generated in this study was made publicly available through European Nucleotide Archive and can be found here: https://www.ebi.ac.uk/ena/browser/text-search?query=PRJEB35029 (accession numbers PRJEB35029, PRJEB44021, accessed on 28 March 2021).

## Supplementary Materials

The following are available online at https://www.mdpi.com/article/10.3390/ijms22094445/s1.

## Abbreviations

AG	Anaerobic germination
bp	Basepairs
bHLH	Basic helix-loop-helix
dai	Days after imbibition
das	Days after sowing
DS	Direct seeding
DEG	Differentially expressed gene
DUF	Domain of unknown function
GEMMA	Genome-wide Efficient Mixed Model Analysis
GLM	Generalized linear model
GWAS	Genome wide association study
HISAT2	Hierarchical indexing for spliced alignment of transcripts 2
Indels	Insertions-deletions
IPP	Isopentenyl pyrophosphate
IRRI	International Rice Research Institute
IRGSP	International Rice Genome Sequencing Project
kb	Kilobasepairs
KHO	Khao Hlan On
LD	Linkage disequilibrium
LOD	Logarithm of the odds
LTP	Lipid transfer protein
Mb	Megabasepairs
MR	Ma-Zhan Red
NILs	Near isogenic lines
PAs	Proanthocyanidins
PAV	Presence-absence variant
PROLM	Prolamin
RIN	RNA integrity number
RNA-Seq	RNA Sequencing
ROS	Reactive Oxygen Species
SNP	Single Nucleotide Polymorphism
Q-Q plot	Quantile-quantile plot
QTL	Quantitative trait locus
TCA	tricarboxylic acid
TTP	Trehalose-6-phosphate phosphatase
3K RG	3000 Rice Genomes
