# Supplementary material for: Enriched-GWAS and Transcriptome Analysis to Refine and Characterize a Major QTL for Anaerobic Germination Tolerance in Rice
Source: Int J Mol Sci. 2021 Apr 24;22(9):4445. doi: 10.3390/ijms22094445 (PMC8123023; doi:10.3390/ijms22094445)

Figure S1. *AG2* was detected in several QTL mapping populations using a variety of AG donor lines. *AG2* detected in a span of ~ 3Mb - 18Mb region in a number of QTL mapping populations, including Ma-Zhan Red (Septiningsih et al. 2013), Khao Hlan On (Angaji et al. 2010) , Kharshu 80A (Baltazar et al. 2019), Nanhi (Baltazar et al. 2014) or TKM9 (Septiningsih et al. unpublished) as donor and either IR42 or IR64 as susceptible parent.

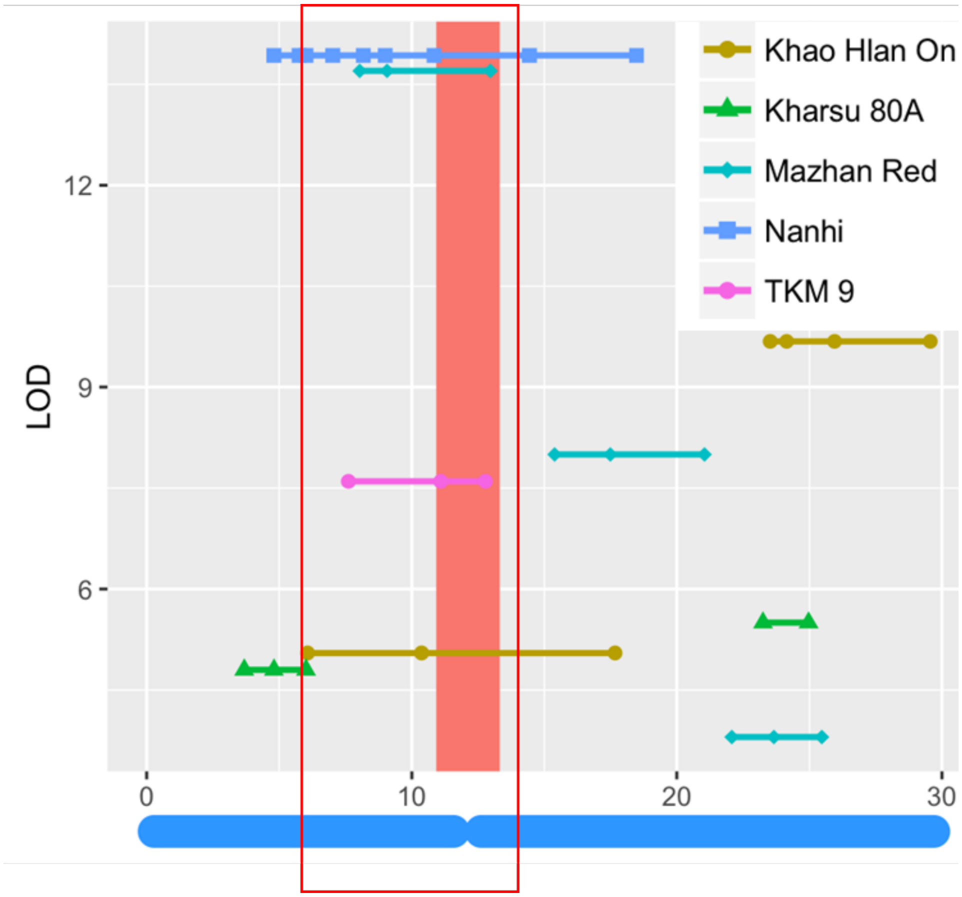

Figure S2. Development of an enriched haplotype of *AG2* GWAS panel. (**a**) Selection of 100 closest relatives to MR from the 3000 genome set based on SNP similarly for each of the 17 bins (Bin1 selection is shown as example). X-axis shows 3000 genomes and y-axis respective SNP congruency with MR haplotype for the 1000 SNP bin. (**b**) *AG2* enriched panel sorted by level of tolerance (tolerant red on top to susceptible green on bottom indicted on the left). The 17 bins for selection are depicted on the right with bins scoring positive for Ma-Zhan Red similarity in black and bins scoring negative for Ma-Zhan Red similarity in white throughout the *AG2* region (6Mb-13Mb).

(a)

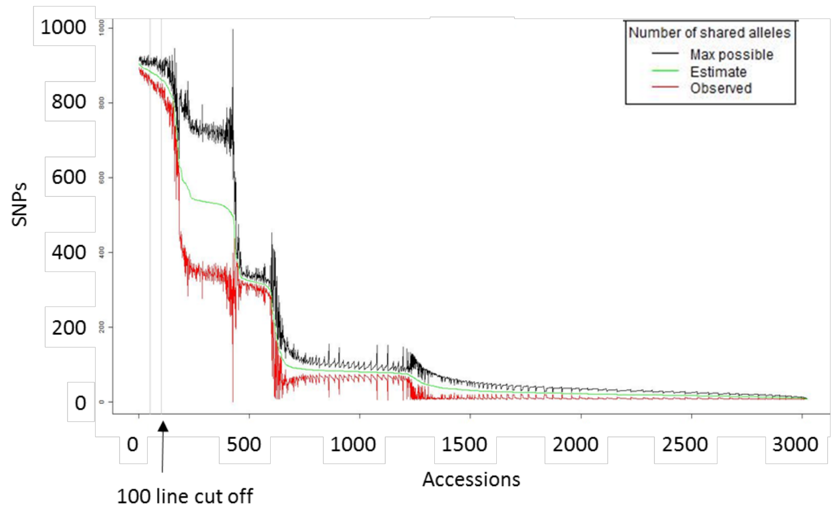

(b)

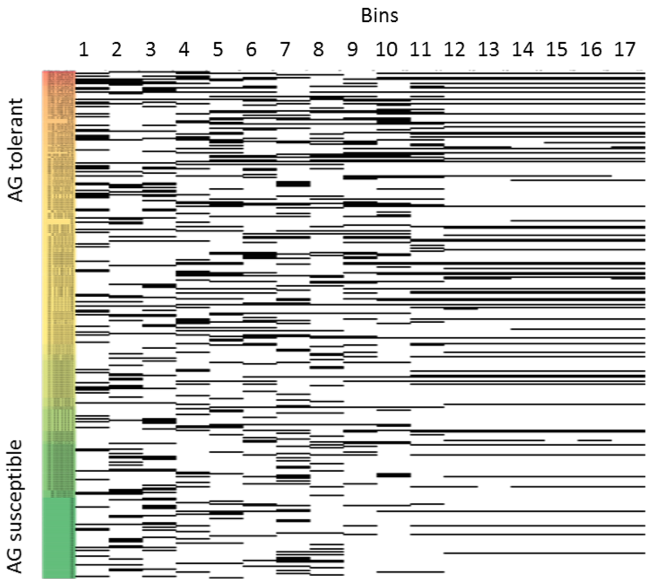

Figure S3. Phylogenetic neighbor-joining tree of the enriched GWAS panel. Subpopulations within the panel are detailed in the upper box at right, shown with lines marked with different colors. Pericarp colors of the seeds used in the panel are detailed in the lower box at right, shown with various shapes in different colors.

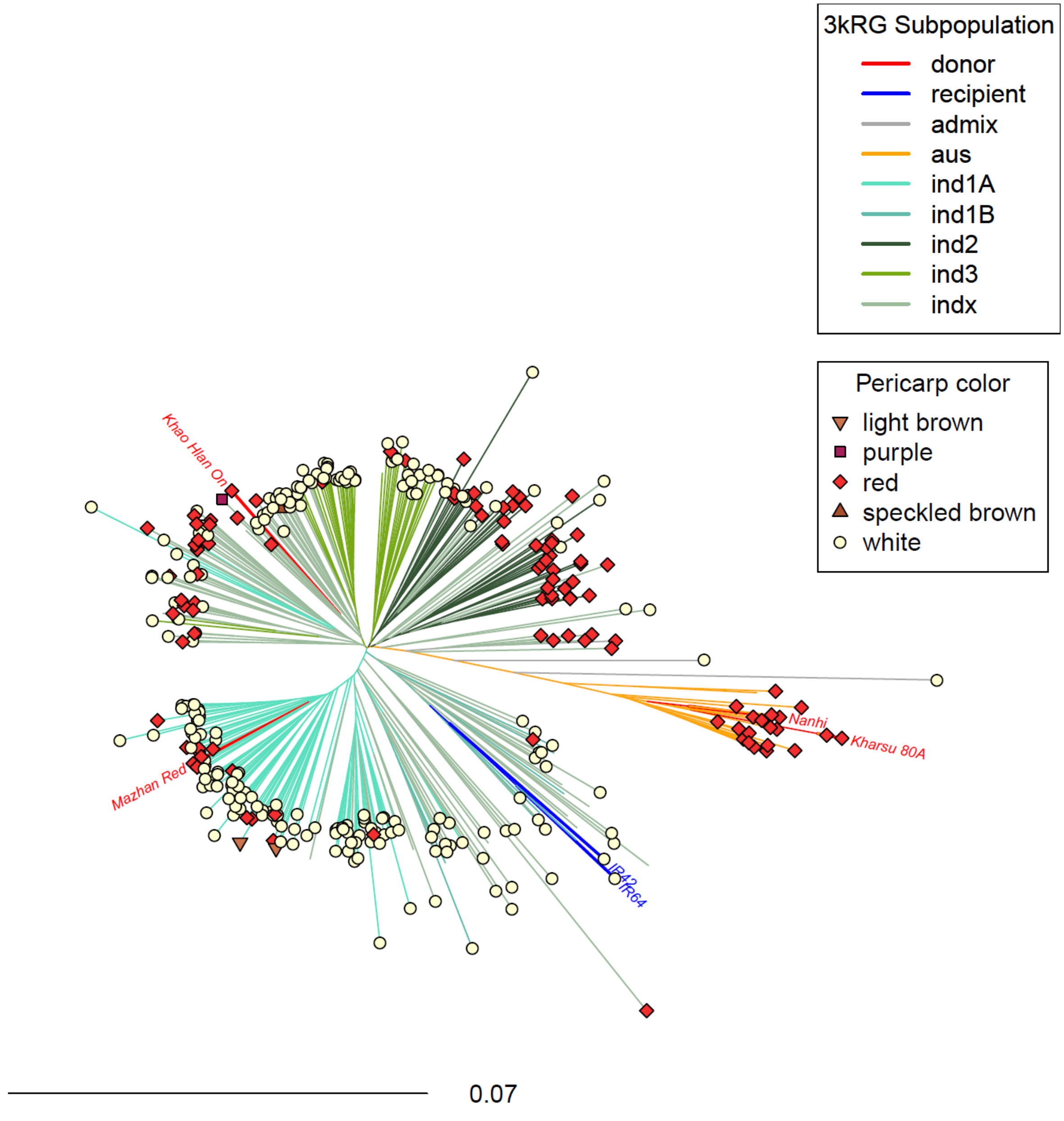

Figure S4. Phenotypic distribution for AG tolerance trait in the enriched *AG2* panel. (**a**) The phenotypic distribution of untransformed data (left) and transformed data (right). The Y axis shows the logit-transformed phenotype (log of odds of survival). Logit of 0 corresponds to 50% chance of survival, and the lowest value of -5.594711 corresponds to having only one sample survived out of 30, and only in one replication out of 3. (**b**) QQ plots of untransformed and transformed data. (**c**) QQ plots of the residuals when the first 5 PCs were added to the model (as in the GWAS). Residuals of untransformed data were not normal (Shapiro-Wilk normality test *p*-value < 2.2e-16), while residuals for the transformed data were within normal variation (Shapiro-Wilk *p*-value = 0.8714).

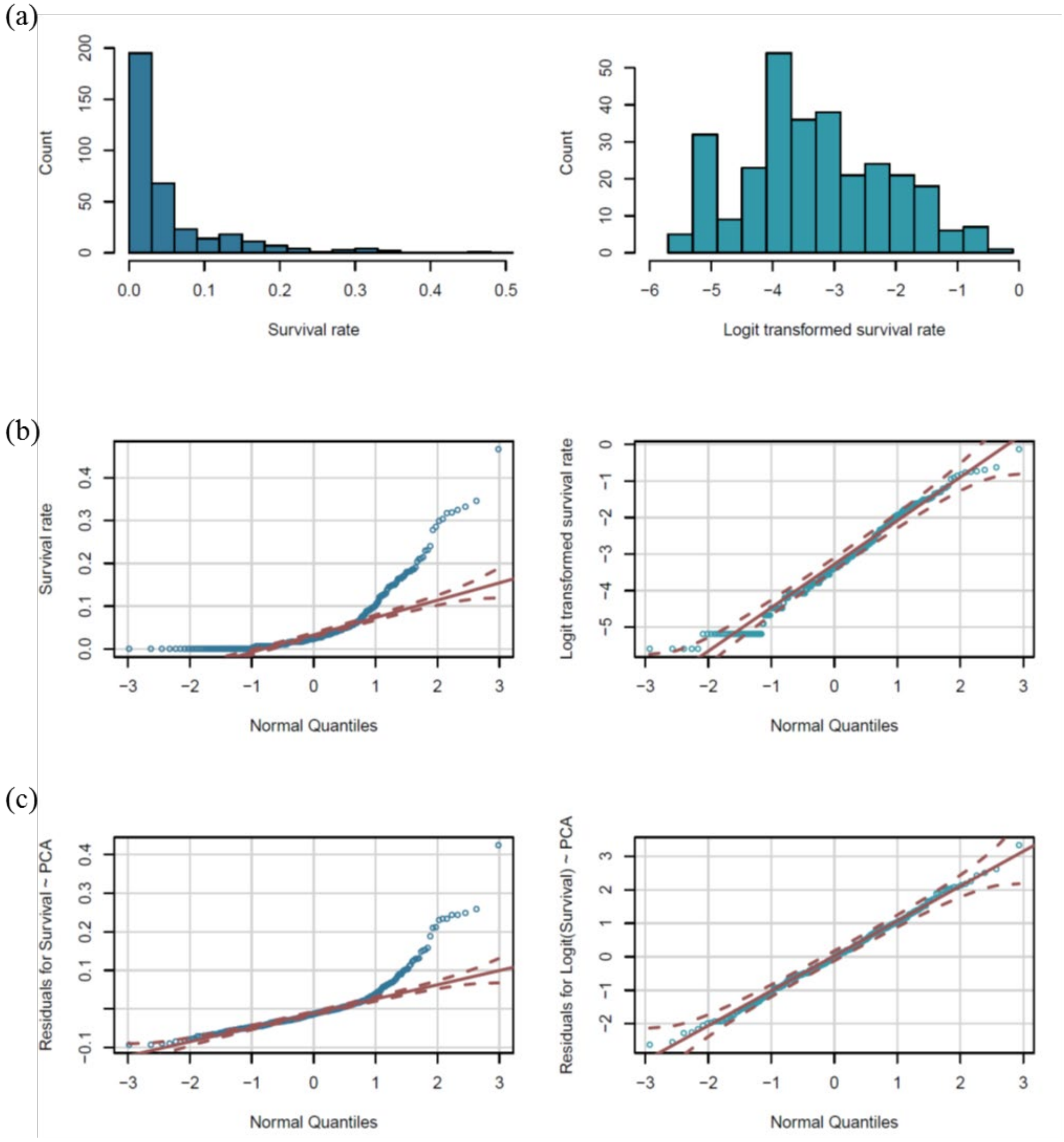

Figure S5. Detection of pericarp color peak on chromosome 7 in the enriched GWAS panel. The Manhattan plot shows the level of significance for SNPs correlated with pericarp color from the GWAS analysis (numbers across the x-axis indicate the chromosome). The QQ plot is shown at the bottom

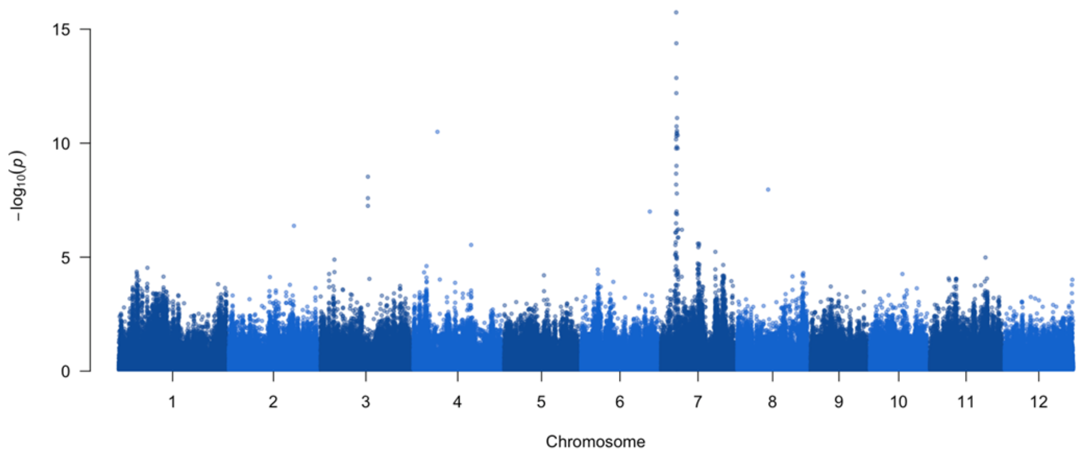

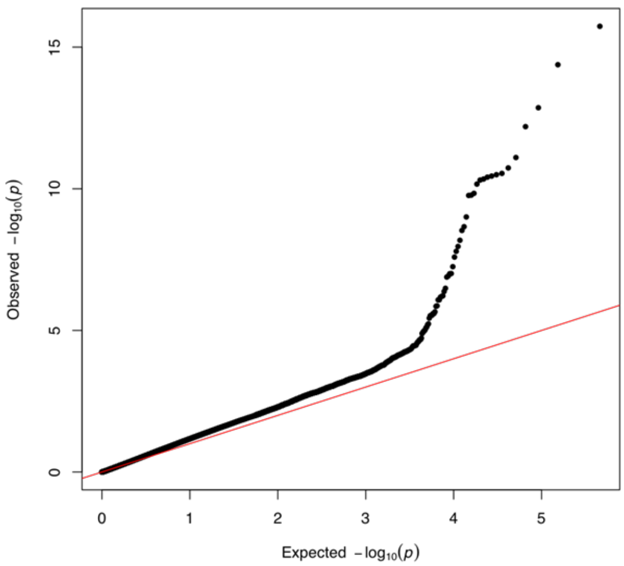

Figure S6. A close-up view of pericarp color peak region on chromosome 7. The Manhattan plot shows the level of significance for SNPs correlated with pericarp color from the GWAS analysis (numbers across the x-axis indicate the chromosome position).

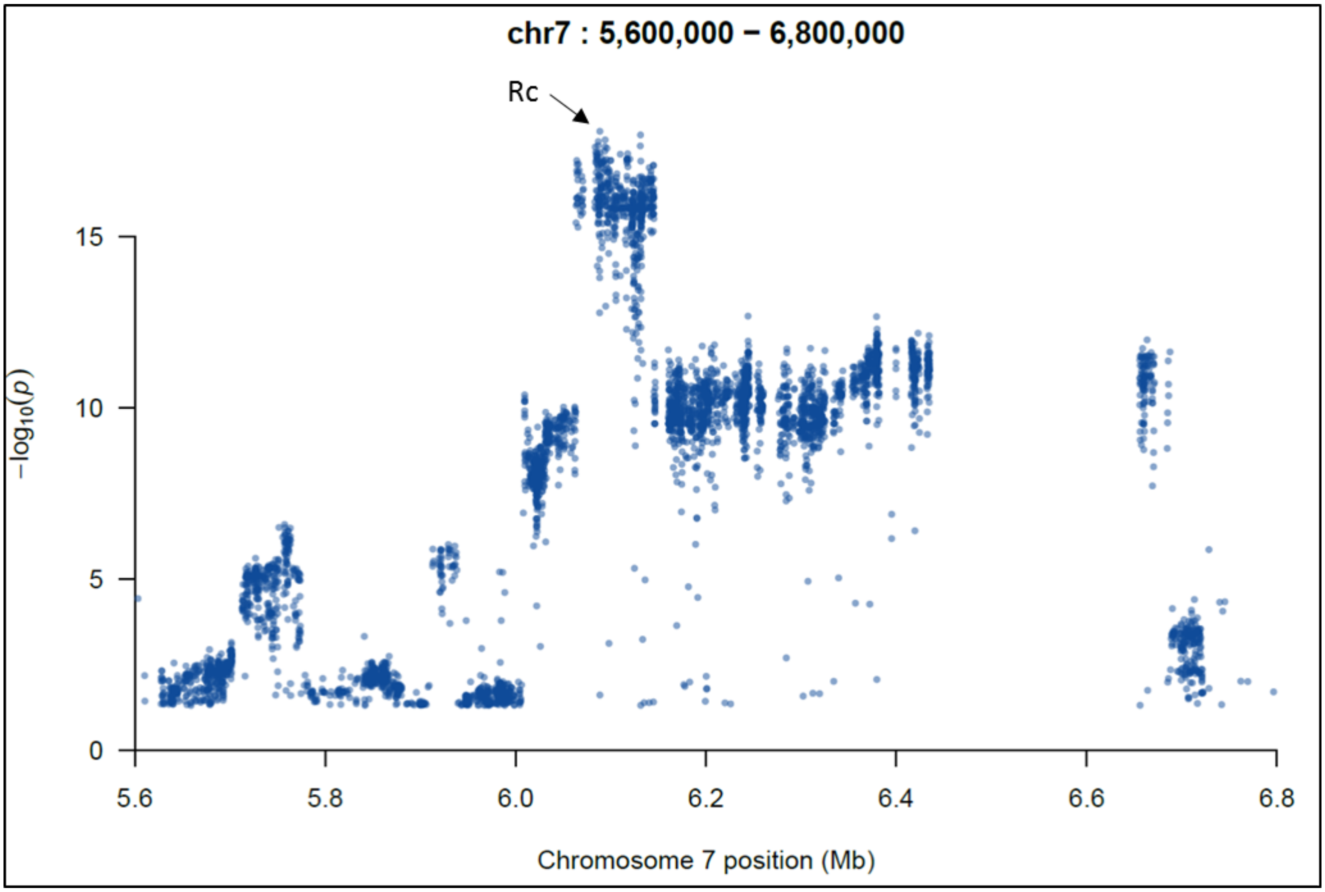

Figure S7. Bean plots of the AG phenotyping data. Bean plots of (**a**) untransformed data and (**b**) logit-transformed data stratified by five major haplotype groups defined by significant SNPs in the region.

(**a**)

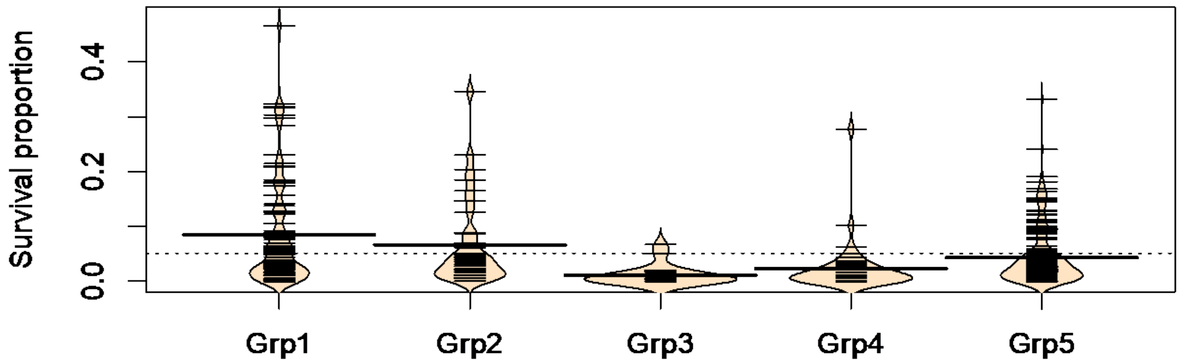

(**b**)

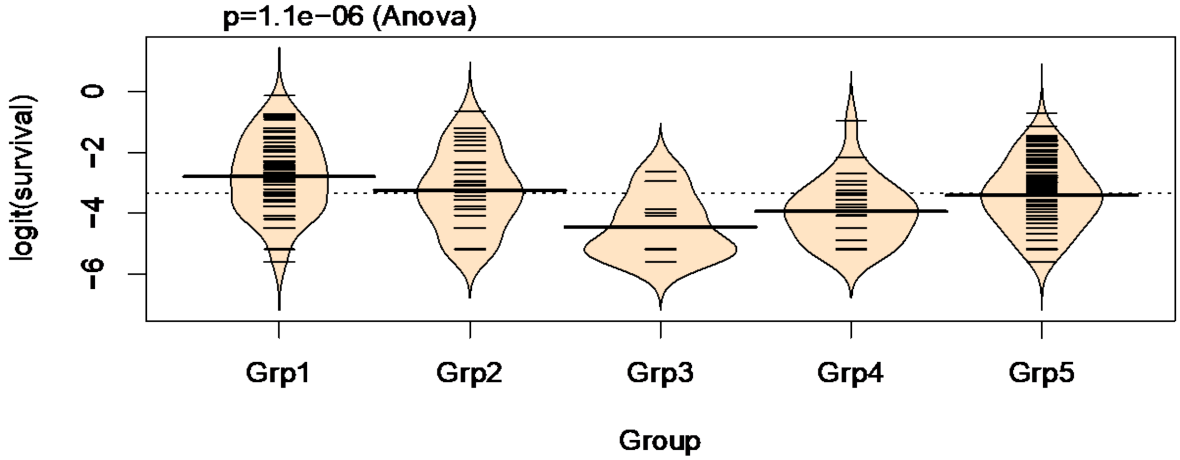

Supplement: Supplementary file 1 [file ijms-22-04445-s001.zip › Supplemetary Figures_17April2021.pdf]
